# Evaluation of the Survival Outcomes of Multiple Myeloma Patients According to Their Plasmacytoma Presentation at Diagnosis

**DOI:** 10.4274/tjh.galenos.2019.2019.0061

**Published:** 2020-11-19

**Authors:** Rafiye Çiftçiler, Hakan Göker, Haluk Demiroğlu, Salih Aksu, Nilgün Sayınalp, İbrahim C. Haznedaroğlu, Ümit Yavuz Malkan, Yahya Büyükaşık, Osman Özcebe

**Affiliations:** 1 Hacettepe University Faculty of Medicine, Department of Hematology, Ankara, Turkey; 2 University of Health Sciences Turkey, Dışkapı Training and Research Hospital, Clinic of Hematology, Ankara, Turkey

**Keywords:** Multiple myeloma, Bone-related plasmacytoma, Soft tissue-related plasmacytoma

## Abstract

**Objective:**

Multiple myeloma (MM) associated with extramedullary (EM) plasmacytoma has a poor therapeutic response and poor outcomes when treated with conventional chemotherapy. EM plasmacytoma is divided into two groups: the first group comprises tumors that are extending directly from osteolytic bone lesions (EM-B, bone-related), while the second results from plasmacytoma infiltration into soft tissues with no relationship to the bone (EM-S, soft tissue-related). This study aimed to compare the general characteristics and survival outcomes of transplant-eligible MM patients who had EM-S or EM-B and MM patients who did not have plasmacytoma at the time of diagnosis.

**Materials and Methods:**

This study was performed in a retrospective manner. The MM patients who were treated at our tertiary care center between January 2003 and January 2017 were evaluated retrospectively for the presence of plasmacytoma at diagnosis.

**Results:**

There were 141 (78.3%) MM patients who did not have plasmacytoma, 22 (12.2%) MM patients who had EM-B, and 17 (9.4%) MM patients who had EM-S at the time of diagnosis in this study. The 5-year overall survival was 63% in patients who had bone EM-B, 63% in patients who had EM-S, and 80% in patients who did not have plasmacytoma (p=0.02). The 5-year disease-free survival was 47% in patients who had EM-B, 35% in patients who had EM-S, and 54% in MM patients who did not have plasmacytoma (p=0.15).

**Conclusion:**

These findings lead us to suggest that MM patients with EM plasmacytoma at the time of diagnosis have poorer prognosis than patients without plasmacytoma, even if autologous stem cell transplantation is performed. The presence of EM involvement negatively affects survival outcomes.

## Abstract (tr)

**Amaç:**

Ekstramedüller (EM) plazmasitom ile ilişkili multipl myelom (MM), geleneksel kemoterapi ile tedavi edildiğinde zayıf bir terapötik yanıta ve kötü sonuçlara sahiptir. EM plazmasitoma iki gruba ayrılır: ilk grup doğrudan osteolitik kemik lezyonlarından uzanan tümörlerden oluşurken, ikincisi yumuşak dokulara plazmasitom infiltrasyonundan, kemikle ilişkisi olmayan plasmasitomdur. Bu çalışma, kemikle ilişkili yada yumuşak doku ile ilişkili olan ve tanı anında plazmasitomu olmayan MM hastalarının genel özelliklerini ve sağkalım sonuçlarını karşılaştırmayı amaçladı.

**Gereç ve Yöntemler:**

Bu çalışma retrospektif olarak yapılmıştır. Ocak 2003-Ocak 2017 tarihleri arasında üçüncü basamak merkezimizde tedavi gören MM hastaları tanı anında plazmasitom varlığı açısından retrospektif olarak değerlendirildi.

**Bulgular:**

Bu çalışmaya plazmositom olmayan 141 (%78,3) MM, kemik plasmasitomu olan 22 (%12,2) ve tanı anında yumuşak doku plasmasitomu olan 17 (%9,4) MM hastası alındı. Beş yıllık genel sağkalım, kemik plasmasitomu olan hastalarda %63, yumuşak doku plasmasitomu olan hastalarda %63 ve plazmositom olmayan hastalarda %80 saptandı (p=0,02). Beş yıllık hastalıksız sağkalım ise kemik plasmasitomu olanlarda %47, yumuşak doku plasmasitomu olanlarda %35 ve plazmasitom olmayan MM hastalarında %54 saptandı (p=0,15).

**Sonuç:**

Sonuç olarak, bu bulgular bizi, tanı anında EM plazmasitomu olan MM hastalarının, kök hücre nakli yapılsa bile, plazmasitomu olmayan hastalara göre kötü prognoza sahip olduğunu düşündürmektedir. EM tutulumunun varlığı sağkalım sonuçlarını olumsuz etkilemektedir.

## Introduction

Multiple myeloma (MM) is defined by the proliferation of neoplastic plasma cells in the bone marrow accompanied by various clinical manifestations including lytic bone lesions, anemia, hypercalcemia, renal function impairment, and recurrent infections [1]. Extramedullary (EM) plasmacytoma is divided into two groups: the first group comprises tumors that are extending directly from osteolytic bone lesions (EM-B, bone-related), while the second results from plasmacytoma infiltration into soft tissues with no relationship to the bone (EM-S, soft tissue-related). EM plasmacytoma can develop in association with MM or as an isolated form. EM plasmacytoma has been reported in 15%-20% of MM patients at the time of diagnosis and develops in 15% of patients during the course of the disease [2]. EM-S plasmacytoma and EM-B plasmacytoma are different in terms of their location, tumor progression, and survival outcomes. EM plasmacytoma that accompanies MM differs from solitary EM plasmacytoma [3].

MM associated with EM plasmacytoma has a poor therapeutic response and poor outcomes when treated with conventional chemotherapy [4]. This study aimed to compare the general characteristics and survival outcomes of MM patients who had EM-S plasmacytoma or EM-B plasmacytoma and MM patients who did not have plasmacytoma at the time of diagnosis.

## Materials and Methods

### Study Design and Data Collection

This study was performed in a retrospective manner. Demographic data, diagnoses, and treatment data of the patients were obtained from the hospital database. As a result of the application standards of the hospitals of Hacettepe Medical School, it has been recognized from the patient records that all of the studied patients had given informed consent at the time of hospitalization and before the administration of chemotherapy and other relevant diagnostic/therapeutic standards of care.

### Patients and Disease Characteristics

The MM patients who were treated at our tertiary care center between January 2003 and January 2017 were evaluated retrospectively for the presence of plasmacytoma at diagnosis. In this study, 21.6% of the 180 patients who underwent autologous stem cell transplantation (ASCT) were patients with plasmacytoma at the time of diagnosis and bone marrow involvement. The patients without plasmacytoma were patients only with bone marrow involvement but no plasmacytoma at the time of diagnosis. The presence of EM disease was diagnosed in most cases by magnetic resonance imaging (MRI), computed tomography (CT) scans, or positron emission tomography (PET), which were carried out whenever an EM spread of disease was suspected on the basis of clinical or radiographic findings. Plasmacytoma was diagnosed by pathological examination in 30 (79.4%) out of 39 patients with plasmacytoma findings on CT, MRI or PET.

The patients who underwent ASCT were divided into three groups of MM with EM-S plasmacytoma, MM with EM-B plasmacytoma, and MM with no plasmacytoma at the time of diagnosis. All cases were included following EM plasmacytoma assessment at diagnosis and no relapse cases were included. All patients underwent ASCT after receiving 6-8 courses of induction chemotherapy. Patients received VCD (bortezomib/cyclophosphamide/dexamethasone), VD (bortezomib/dexamethasone), or VAD (vincristine, doxorubicin, and dexamethasone) as induction therapy. Patients who were not eligible for transplantation and patients who received more than one ASCT were excluded from the study. Response was determined according to the current International Myeloma Working Group response criteria [5]. Cytogenetic data were available only in a minority of cases and were not considered in this analysis.

### Statistical Analysis

Statistical analyses were performed using SPSS 25. The variables were investigated using visual (histograms, probability plots) and analytical methods (Kolmogorov-Smirnov/Shapiro-Wilk test) to determine whether they were normally distributed or not. One-way ANOVA was used to compare parameters using means and standard deviations for normally distributed variables, while the Kruskal-Wallis test was used to compare parameters for non-normally distributed variables. Survival analyses were performed using the Kaplan-Meier test. Multivariate analysis of predictors of survival was performed using the Cox regression test. Parameters with p≤0.15 in univariate tests were included in the multivariate analysis, and p<0.05 was considered to indicate statistical significance.

## Results

### Patient Characteristics

A total of 180 MM patients who underwent ASCT were included in the study between 2003 and 2017. Patient characteristics are summarized in Table 1. There were 141 (78.3%) patients who did not have plasmacytoma, 22 (12.2%) patients who had EM-B plasmacytoma, and 17 (9.4%) patients who had EM-S plasmacytoma at the time of diagnosis. There were 113 (62.8%) male and 67 (37.2%) female patients with a median age of 57 (range: 35-72) years at the time of diagnosis. The numbers of patients classified with Eastern Cooperative Oncology Group performance status (ECOG PS) 0, 1, 2, and 3 at diagnosis were 27 (15.0%), 86 (47.8%), 55 (30.6%), and 12 (6.7%), respectively. There was no statistically significant difference between the three groups in terms of ECOG PS (p=0.13) [6]. Ninety-five (52.8%) patients received VAD, 51 (28.3%) patients received VCD, and 34 (18.9%) patients received VD as induction chemotherapy (p=0.29). No statistically significant difference was found between the three groups in terms of sex (p=0.36). The ISS staging (p=0.35) of all three groups was similar. Serum hemoglobin (Hb) (p=0.43), platelet  count (p=0.25), calcium (p=0.72), lactate dehydrogenase (LDH) (p=0.32), and creatinine level (p=0.88) at diagnosis showed no statistically significant differences between the three groups. Lytic bone lesions were more common with statistical significance in patients who had EM-B or EM-S plasmacytoma (p=0.004) than in patients who did not have plasmacytoma. Radiotherapy was performed for more patients who had EM plasmacytoma than patients who did not have plasmacytoma (p<0.001). There was no statistically significant difference between the three groups in terms of MM types (p=0.64). Sites involved were soft tissues surrounding the axial skeleton in 76.4% (n=13 patients) of cases. Plasmacytomas of the breast (n=1, 5.9%), spleen (n=1, 5.9%), oral cavity (n=1, 5.9%), and skin (n=1, 5.9%) accounted for 23.5% of cases. EM-B plasmacytomas were located in the vertebrae (n=14, 63.6%), ribs (n=1, 4.5%), sternum (n=2, 11.7%), clavicle (n=2, 11.7%), and pelvis (n=3, 13.6%). The median number of involved sites of plasmacytoma was 1 (range: 1-5) for patients who had EM plasmacytoma.

Disease status after induction chemotherapy was similar between the three groups (p=0.41). However, disease status after ASCT was better in patients without plasmacytoma than in patients with EM-B and EM-S plasmacytoma (p=0.002). Relapse rate (p=0.01) and mortality rate (p<0.001) were higher with statistical significance in patients who had EM-B or EM-S plasmacytoma than in patients who did not have plasmacytoma.

### Overall Outcomes

**Figure 1:**
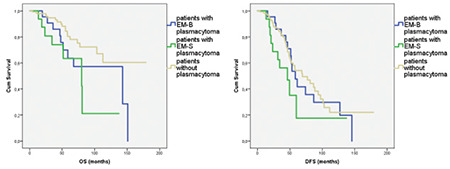
Overall survival (OS) (p=0.02) and disease-free survival (DFS) (p=0.15) of patients according to their plasmacytoma presentation at diagnosis.

The median follow-up period was 39.3 months (range: 4.2-178.9 months) for the entire group. The 3-year overall survival (OS) was 85% in patients who had EM-B plasmacytoma, 74% in patients who had EM-S plasmacytoma, and 95% in MM patients who did not have plasmacytoma. The 5-year OS was 63% in patients who had EM-B plasmacytoma, 63% in patients who had EM-S plasmacytoma, and 80% in patients who did not have plasmacytoma (p=0.02) (Figure 1).

The 3-year disease-free survival (DFS) was 81% in patients who had EM-B plasmacytoma, 56% in patients who had EM-S plasmacytoma, and 81% in patients who did not have plasmacytoma. The 5-year DFS was 47% in patients who had EM-B plasmacytoma, 35% in patients who had EM-S plasmacytoma, and 54% in patients who did not have plasmacytoma (p=0.15) (Figure 1).

**Figure 2:**
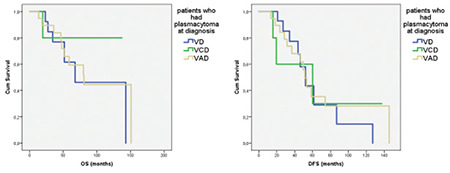
Overall survival (OS) (p=0.89) and disease-free survival (DFS) (p=0.82) according to induction chemotherapy in patients who had EM plasmacytoma at diagnosis.

The 3-year OS was 76% in patients with EM plasmacytoma at diagnosis who received VD, 80% in patients who received VCD, and 83% in patients who received VAD as induction chemotherapy. The 5-year OS was 61% in patients with EM plasmacytoma at diagnosis who received VD, 80% in patients who received VCD, and 59% in patients who received VAD as induction chemotherapy (p=0.89) (Figure 2).

The 3-year DFS was 76% in patients with plasmacytoma at diagnosis who received VD, 60% in patients who received VCD, and 78% in patients who received VAD as induction chemotherapy. The 5-year OS was 29% in patients with plasmacytoma at diagnosis who received VD, 30% in patients who received VCD, and 35% in patients who received VAD as induction chemotherapy (p=0.82) (Figure 2).

### Cox Regression Analysis

In univariate analyses the factors that affected OS were age of the patient (≤57 years) (p=0.05) and absence of plasmacytoma at diagnosis (p=0.01), as shown in Table 2. Cox regression analysis revealed absence of plasmacytoma at diagnosis (p=0.01) as the only parameter to predict OS.

In univariate analyses the factors that affected DFS were age of the patient (≤57 years) (p=0.01), receiving radiotherapy (p=0.05), and ISS stage of the disease (p=0.12). Cox regression analysis revealed age of the patient (≤57 years) (p=0.03) as the only parameter to predict DFS.

## Discussion

Focal infiltration by monoclonal plasma cells in the absence of systemic disease can be observed as solitary plasmacytoma. EM plasmacytoma can also develop with systemic disease [1]. EM plasmacytoma has been defined to occur in up to 15%-20% of MM patients at the time of diagnosis. Additionally, it develops in 15% of patients during the course of the disease [7]. In this study, the survival outcomes of MM patients who underwent ASCT were evaluated according to their plasmacytoma presentation at diagnosis. Plasmacytoma was detected at the time of diagnosis in 21.6% of all patients. While 12.2% of the patients had EM-B plasmacytoma, 9.4% of them had EM-S plasmacytoma. This study showed that patients who did not have any plasmacytoma had better OS than patients who had EM-B or EM-S plasmacytoma. In multivariate analyses, the only parameter predicting OS was the absence of plasmacytoma at diagnosis. Additionally, DFS was better in patients without plasmacytoma than patients with EM plasmacytoma. However, there was no statistically significant difference in DFS for all three groups.

In time-dependent analyses, Varettoni et al. showed that the presence of EM involvement at any time during the course of disease is associated with shorter OS and DFS, even after adjusting for age, sex, and stage [8]. MM with EM plasmacytoma showed significant differences from the rest of the MM population regarding age, sex, MM subtype, disease stage, and prior history of monoclonal gammopathy of unknown significance. In addition, patients who developed EM spread during follow-up showed significantly lower Hb and M-protein and higher LDH levels compared with patients with EM disease at diagnosis [8]. Another study reported that in patients with MM with EM-B and EM-S plasmacytoma, the disease had an aggressive course, with a median OS of 15 months [9].

There are few studies focusing on treatment of MM patients with EM disease. Some clinical reports indicated a low efficacy of thalidomide on EM disease [10,11], while bortezomib seems more promising in this setting [12,13]. Wu et al. [14] evaluated the outcomes of newly diagnosed MM with and without EM plasmacytomas and reported that the presence of EM plasmacytomas at diagnosis was associated with poor prognosis in patients treated with conventional chemotherapy. However, patients treated with high-dose melphalan followed by ASCT had similar outcomes, regardless of the presence or absence of EM plasmacytomas [14]. Lee et al. [15] showed that the negative impact of EM plasmacytomas was significant on OS (p=0.007) and nearly significant on DFS (p=0.054) among patients not eligible for ASCT. On the other hand, some studies showed that there was no statistically significant difference in survival outcomes in patients with MM with or without EM plasmacytoma at diagnosis who received ASCT after chemotherapy. These studies reported that ASCT can succeed in dealing with the negative prognostic effect of EM plasmacytomas at the time of diagnosis of MM [8,16]. In this study, although all patients underwent ASCT, the survival outcomes of patients with EM were worse than those of patients without EM plasmacytoma at diagnosis. When we look only at patients with EM plasmacytoma, there was no significant difference in OS and DFS between VAD, VCD, or VD as induction chemotherapy.

Our study had a few limitations. The lack of data regarding the cytogenetic features of the patients is the major limitation of this study. Additionally, all patients did not receive the same chemotherapy before ASCT. In our study, as in other studies, it is clear that patients with EM plasmacytoma have a poor prognosis. In the era of highly active new anti-myeloma regimens, further trials are needed to determine the effect of MM presenting with EM plasmacytoma, preferably with higher patient numbers and longer follow-up.

## Conclusion

These finding lead us to suggest that MM patients who have EM plasmacytoma at the time of diagnosis have a poorer prognosis than patients without plasmacytoma, even if ASCT is performed. The presence of EM involvement negatively affects survival outcomes.

## Figures and Tables

**Table 1:**
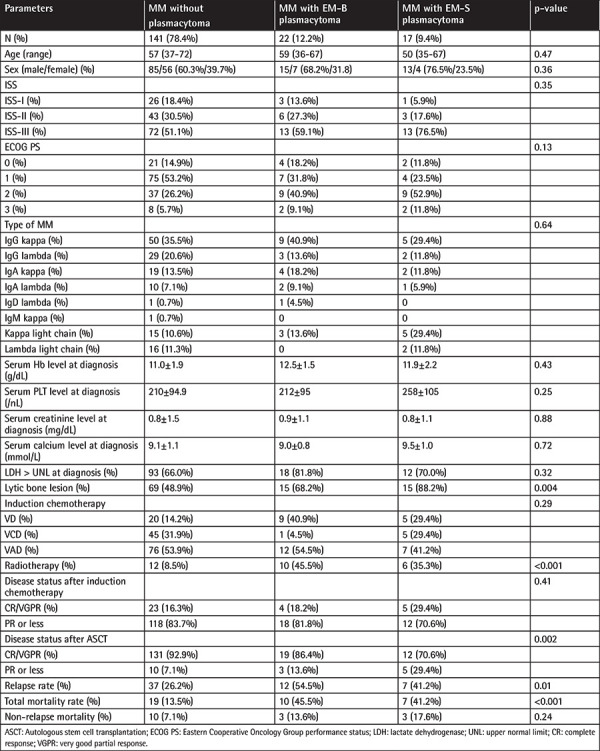
Baseline clinical and demographic characteristics of patients with MM.

**Table 2:**
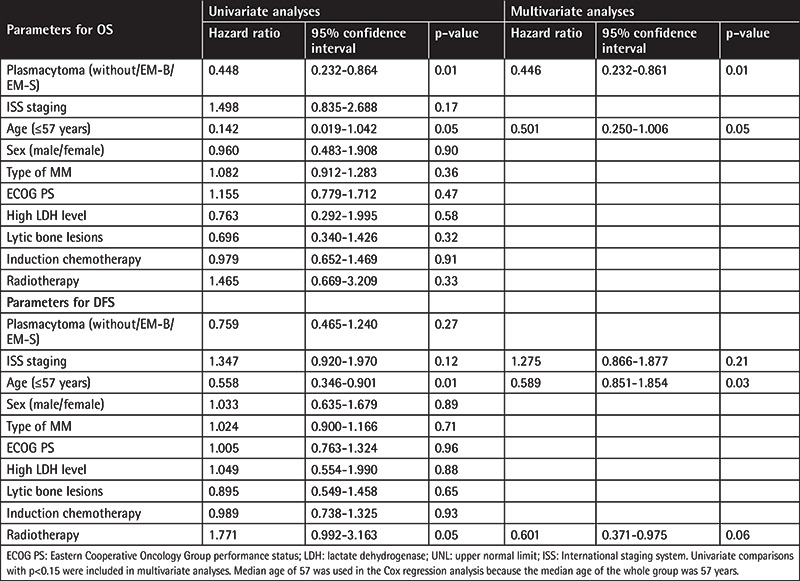
Univariate and multivariate analyses (Cox model) of overall survival (OS) and disease-free survival (DFS) for all patients.
